# Black Phosphorus Quantum Dots Enhance the Radiosensitivity of Human Renal Cell Carcinoma Cells through Inhibition of DNA-PKcs Kinase

**DOI:** 10.3390/cells11101651

**Published:** 2022-05-16

**Authors:** Yue Lang, Xin Tian, Hai-Yue Dong, Xiang-Xiang Zhang, Lan Yu, Ming Li, Meng-Meng Gu, Dexuan Gao, Zeng-Fu Shang

**Affiliations:** 1 State Key Laboratory of Radiation Medicine and Protection, School of Radiation Medicine and Protection, Medical College of Soochow University, Collaborative Innovation Center of Radiation Medicine of Jiangsu Higher Education Institutions, Soochow University, Suzhou 215123, China; langyue2080@163.com (Y.L.); xtian@suda.edu.cn (X.T.); 20194220012@stu.suda.edu.cn (H.-Y.D.); zhangxxszu@sina.com (X.-X.Z.); lim1984@suda.edu.cn (M.L.); 2 Department of Nuclear medicine, The Second Affiliated Hospital of Xuzhou Medical University, Xuzhou 221000, China; 3 Suzhou Digestive Diseases and Nutrition Research Center, The Affiliated Suzhou Hospital of Nanjing Medical University, Suzhou 215008, China; yulan_229@163.com; 4 Department of Nuclear Medicine, The Affiliated Suzhou Hospital of Nanjing Medical University, Suzhou 215002, China; 5 Department of Urology, Shandong Provincial Hospital Affiliated to Shandong First Medical University, Jinan 250021, China

**Keywords:** black phosphorus quantum dots, renal cell carcinoma, irradiation, DNA-PKcs, DNA damage repair

## Abstract

Renal cell carcinoma (RCC) is one of the most aggressive urological malignancies and has a poor prognosis, especially in patients with metastasis. Although RCC is traditionally considered to be radioresistant, radiotherapy (RT) is still a common treatment for palliative management of metastatic RCC. Novel approaches are urgently needed to overcome radioresistance of RCC. Black phosphorus quantum dots (BPQDs) have recently received great attention due to their unique physicochemical properties and good biocompatibility. In the present study, we found that BPQDs enhance ionizing radiation (IR)-induced apoptotic cell death of RCC cells. BPQDs treatment significantly increases IR-induced DNA double-strand breaks (DSBs), as indicated by the neutral comet assay and the DSBs biomarkers γH2AX and 53BP1. Mechanistically, BPQDs can interact with purified DNA–protein kinase catalytic subunit (DNA-PKcs) and promote its kinase activity in vitro. BPQDs impair the autophosphorylation of DNA-PKcs at S2056, and this site phosphorylation is essential for efficient DNA DSBs repair and the release of DNA-PKcs from the damage sites. Consistent with this, BPQDs suppress nonhomologous end-joining (NHEJ) repair and lead to sustained high levels of autophosphorylated DNA-PKcs on the damaged sites. Moreover, animal experiments indicate that the combined approach with both BPQDs and IR displays better efficacy than monotreatment. These findings demonstrate that BPQDs have potential applications in radiosensitizing RCC cells.

## 1. Introduction

Renal cell carcinoma (RCC) is a frequently lethal urological disease and accounts for ~90% of kidney cancers [1]. Its incidence rate has increased during the previous decade, and one-third of newly diagnosed cases have multiple distant metastases. The metastatic spread of cancer cells renders RCC incurable by surgical resection and decreases the 5-year survival rate to approximately 8% [2]. Palliative radiotherapy (RT) plays a valuable role in the management of metastatic RCC, especially for brain and painful bone metastasis [3]. Radioresistance remains the major obstacle to limit the efficacy of RT, and there is an urgent need to develop novel treatment strategies to increase RCC cell radiosensitivity. DNA is the principle cellular target for the biological effects of ionizing radiation (IR). The most lethal forms of DNA lesions caused by IR are DNA double-strand breaks (DSBs), as a single DSB is sufficient to induce cell cycle arrest or trigger cell death [4]. There are two main pathways to repair DNA DSBs in higher eukaryotes: homologous recombination (HR) and nonhomologous end joining (NHEJ). HR repairs DSBs that are restricted to the late S or G_2_ phases of the cell cycle when a sister chromatid is available [5]. NHEJ is the predominant repair mechanism and may occur throughout all cell cycle phases. NHEJ directly relegates the broken DNA ends following limited DNA processing. The key effector of NHEJ is the DNA-PK complex consisting of DNA-dependent kinase catalytic subunit (DNA-PKcs) and the Ku70/80 heterodimer [6]. Once DNA DSBs occur, Ku70/80 binds rapidly to free DNA ends and recruits DNA-PKcs, which stimulates the activity of DNA-PKcs. The DNA-PK subunits are required for the recruitment of other repair proteins, including DNA ligase IV and XRCC4 [7]. Activated DNA-PKcs can phosphorylate itself and Artemis. Phosphorylated Artemis is activated, and its nuclease activity is implicated in end-processing, whereas autophosphorylation of DNA-PKcs destabilizes its interaction with the DNA ends, and allows subsequent gap-filling or ligation steps [8]. Recent studies have implicated the potential role of DNA-PKcs in cancer development. Overexpression of DNA-PKcs is frequent in a variety of cancers, including RCC [9]. Given its critical role in NHEJ, DNA-PKcs has been recognized as a promising therapeutic target in concert with DNA-damaging agents [10].

Nanoparticles (NPs) are materials with overall dimensions less than 100 nm. Owing to their unique physicochemical properties, NPs have emerged as important players in biomedical fields [11,12], such as antimicrobials, disease diagnosis, drug delivery, cancer photodynamic therapy (PDT), and photothermal therapy (PTT) [13]. Recently, black phosphorus quantum dots (BPQDs) have received great attention due to their good biocompatibility, high specific surface area, and drug loading rate [14]. BPQDs efficiently convert near-infrared (NIR) light into thermal energy and significantly induce cancer-cell-killing effect [14,15]. BPQDs can be used as a gene delivery system for cancer treatment. Polyelectrolyte-polymer-functionalized BPQDs efficiently deliver lysine-specific demethylase 1 small-interfering RNA (siRNA) into PA-1 cells [16]. A recent study incorporated BPQDs into a liposomal bilayer to generate an NIR-light-controlled drug delivery system. This system exhibited excellent cancer-cell-killing effects through rapid intracellular doxorubicin release and photothermal-mediated increased cell membrane permeability, thus easily entering doxorubicin into cell nuclei [17]. BPQDs can also act as radiosensitizers by generating singlet oxygen (^1^O_2_) in tumor in response to X-ray irradiation [18]. The direct intrinsic interaction of BPQDs with cell organelles or specific biomolecules has been reported. Shao et al. revealed that BPQDs directly bind with polo-like kinase 1 (PLK1), through which destabilize mitotic centrosomes [19]. Our previous study showed that BPQDs suppress histone deacetylase 1 (HDAC1) activity and impair HDAC1-mediated deacetylation of the mitotic spindle protein Eg5 in RCC cells, thus disrupting the mitotic spindle and leading to mitotic arrest. In this study, we found that BPQDs slow DNA damage repair in response to X-ray irradiation in RCC 786-O cells. Mechanistically, BPQDs impaired NHEJ repair by directly interacting with DNA-PKcs and suppressing its kinase activity.

## 2. Materials and Methods

### 2.1. Western Blot

The 786-O and A498 cells were cultured with PBS or 20 μg/mL BPQDs for 12 h and then exposed to 5 or 10 Gy X-ray. Protein extraction from 786-O cells was performed, as described preciously [20]. Then, proteins were subjected to 8% SDS-PAGE and transferred onto PVDF membranes. Blots were blocked with 5% non-fat dry milk with primary antibodies at 4 °C overnight, followed by secondary antibodies, and detected by a chemiluminescence imager (Tanon, Shanghai, China). The antibodies used for Western blotting were: Cleaved PARP (CST, 5625, 1:1000), Actin (Beyotime, AA128, 1:1000), DNA-PKcs-ps2056 (Abcam, ab18192, 1:1000), DNA-PKcs (Abcam, ab32566, 1:1000), Chk2-pT68 (CST, 2661S, 1:1000), and Chk2 (CST, 2662S, 1:1000).

### 2.2. Immunofluorescence Assay

For immunofluorescence analysis, cells were cultured on 35 mm^2^ dishes with coverslips, and treated with BPQDs and indicated dose of irradiation. Cells were fixed with 4% paraformaldehyde, and permeabilized in 0.5% Triton X-100 for additional 20 min, and blocked in 5% bovine serum albumin. The samples were incubated with the following antibodies: γH2AX (Millipore, 05-636, 1:1000), 53BP1 (Novusbio, NB100-904, 1:200), DNA-PKcs-ps2056 (Abcam, ab18192, 1:1000), Alexa-488-conjugated anti-rabbit secondary antibodies (CST, 4916, 1:1000), and Alexa-488-conjugated anti-mouse secondary antibodies (CST, 4408, 1:1000).

### 2.3. Flow Cytometry

The 786-O cells were culture onto 60 mm^2^ dishes. Cells were exposed to 20 μg/mL BPQDs along or in combination with 5 Gy IR for 24 h. Cells were harvested, centrifuged, and resuspended. Then, PE-Annexin V and 7-AAD were incubated with cell suspension in the dark condition. Cells were subjected to flow cytometry analysis using a flow cytometer (FACSVerse, BDBiosciences, San Diego, CA, USA).

### 2.4. Comet Assay for DNA Double-Strand Breaks

Cells were treated with 20 μg/mL of BPQDs for 12 h, and washed out with PBS, and were then irradiation with 4 Gy of X-ray. A duration of 4 h post IR, the cells were mixed with low melting point agarose, spread on a comet assay slide. Those slides were left into 4 °C for drying, and incubated with neutral lysis buffer and subjected to electrophoresis. Cells were stained with SYBR Green I, and comet tails were visualized using a confocal microscope (FV1200, Olympus, Tokyo, Japan). Experiments were performed at least three times for each sample.

### 2.5. NHEJ Repair Efficiency

The 786-O cells were cultured in 6-well plates, and 3 μg of CRISPR/Cas9 plasmid was co-transfected with 25 pmol dsODN into 786-O cells. BPQDs (20 μg/mL or 40 μg/mL) were added to the cells 24 h post transfection. Then, the genomic DNA of 786-O cells was extracted by a Genomic DNA Kit (Tiangen, Beijing, China) and real-time PCR (Applied Biosystems, Foster City, CA) was performed.

### 2.6. Animal Experiment

The 1.5-month-old male BALB/c nude mice (Shanghai SLAC Laboratory Animal Co. Ltd., Shanghai, China) were raised in the SPF-level laboratory animal room of Soochow University. A total of 1 × 10^7^ 786-O cells were injected subcutaneously into the flanks of nude mice. When the tumor grew to 50–100 mm^3^, the mice were treated with PBS or BPQDs (1 mg/kg), 10 Gy X-rays or BPQDs + X-rays. Radiation was administered (2 Gy/min) to the tumor xenografts in mice by a linear accelerator (Varian). BPQDs were administered to the tumors on days 0, 3, 6, and 9. Every other day, the volume of tumors was measured and recorded. Mice were sacrificed and the tumor tissues were harvested and fixed in tissue fixation fluid on day 20.

### 2.7. Statistical Analyses

All results are presented as the mean ± standard deviation (s.d.). Comparisons were evaluated by Student’s *t*-test for differences between two groups and ANOVA for differences among three or more groups.

## 3. Results

### 3.1. Synthesis and Characterization and BPQDs

The BPQDs used in this study were prepared according to our previous study [20]. The morphology of the obtained BPQDs is shown in the supporting information. BPQDs have an average diameter of approximately 10 nm and a thickness of 3–6 nm (Figure S1). In addition, the surface zeta potential and hydrodynamic size of BPQDs were measured and are summarized in Table S1.

### 3.2. BPQDs Increase Radiation-Induced Apoptosis of RCC

**Figure 1:**
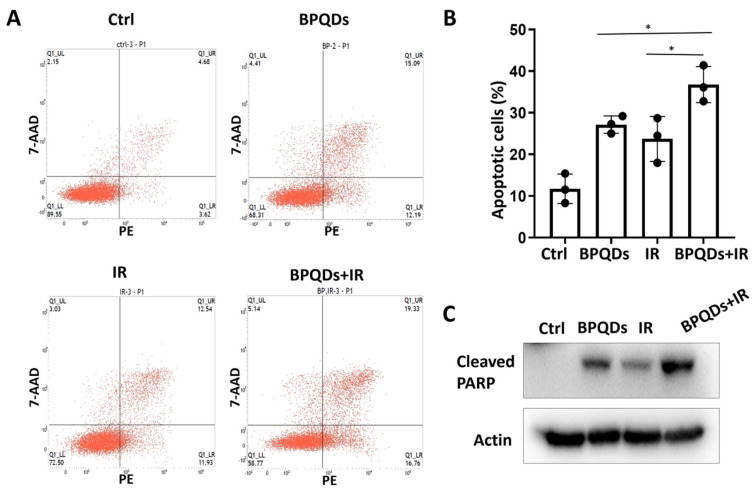
BPQDs enhance IR-induced apoptotic cell death of 786-O cells. (**A**) Cells were exposed to 20 μg/mL BPQDs along or in combination with 5 Gy IR for 24 h, and representative flow cytometry plots; (**B**) the percentage of AnnexinV-positive apoptotic cells (* *p* < 0.05, BRQDs + IR versus BPQDs and IR monotreatment groups). (**C**) Cells were exposed to 20 μg/mL BPQDs along or in combination with 5 Gy IR for 24 h and subjected to immunoblotting with anti-Cleaved PARP (Asp214), and anti-actin antibodies.

Our previous study showed that BPQDs enhance the chemosensitivity of RCC cells by impairing spindle assembly [20]. Spindle-targeting drugs have been proven to be highly active as radiosensitizers to enhance the killing effect of tumor cells and improve clinical outcome for patients with cancers. Here, we determined the potential role of BPQDs in RCC 786-O cell radiosensitivity by treating 786-O cells with IR alone or in combination with BPQDs. The percentage of apoptotic cells was significantly higher in BPQDs- and radiation-treated cells than in either monotreatment group (Figure 1A,B). An increase in combination-treatment-induced apoptosis was also evidenced by cleavage of PARP-1 through immunoblotting analysis (Figure 1C). In addition, we found that BPQDs decrease the capacity of DNA DSBs and enhance IR-induced apoptosis in another RCC cell line, A498 cells (Figures S2 and S3). These results suggest that BPQDs dramatically enhance radiation-induced apoptotic cell death of RCC.

### 3.3. BPQDs Enhance IR-Induced DNA Damage and Slow Damage Repair

**Figure 2:**
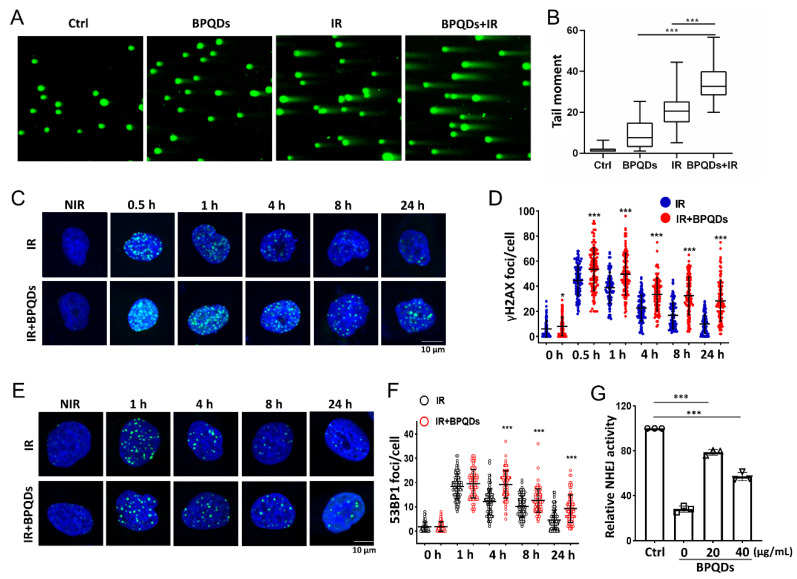
BPQDs decrease the capacity of DNA DSBs in 786-O cells. The 786-O cells were either exposed to 20 μg/mL BPQDs, 4 Gy X-ray irradiation, or combined treatment, and the cells were subjected to neutral single cell gel electrophoresis 4 h posttreatment. (**A**) Representative comet images of different groups of cells. (**B**) Repair ability of DNA DSBs were measured (more than 100 cells were counted, three independent assays). (**C**) Representative IF images showing γH2AX foci in 786-O cells treated with 20 μg/mL BPQDs or PBS 2 h before 2 Gy IR (scale bar = 10 μm). (**D**) Number of γH2AX foci per cell at the indicated time points post-IR (data were generated from three independent experiments. * *p* < 0.05, *** *p* < 0.001). (**E**) Representative IF images showing 53BP1 foci in 786-O cells treated with 20 μg/mL BPQDs or PBS 2 h before 2 Gy IR (scale bar = 10 μm). (**F**) Number of 53BP1 foci per cell at the indicated time points post-IR (data were generated from three independent experiments. *** *p* < 0.001). (**G**) The 786-O cells were transfected with 3 μg Cas9/sgHPRT plasmid and 25 pmol dsODN. At 24 h post-transfection, real-time PCR analysis was performed to measure the dosage of BPQDs that had suppressive effects on NHEJ repair. Nu7441 was adopted as a positive control.

DNA is the principal target of IR in cells [21]. To understand how BPQDs contribute to the IR-induced cell-killing effect on 786-O cells, a comet assay was used to detect DNA DSBs after treatment of 786-O cells with IR along or in combination with BPQDs. The comet tails of cotreated 786-O cells were much longer than those in either mono-treated cells (Figure 2A,B). The phosphorylation of H2AX on its Ser139 (γH2AX) will occur around DNA DSBs and is a sensitive molecular marker of DNA DSBs. Immunofluorescence staining for γH2AX foci was adopted, and showed that BPQDs-treated 786-O and A498 cells had prolonged repair kinetics compared to the mock group of 786-O cells at 0.5–24 h post 2 Gy of IR (Figure 2C,D and Figure S3). An early step in DNA DSBs repair involves the recruitment of 53BP1 to form foci at the damaged DNA ends. The DNA DSBs repair kinetics were also supported by counting 53BP1 foci numbers. We observed a significantly slower rate of DNA DSBs repair in combined-treated 786-O cells than in either BPQDs or IR mono-treated cells (Figure 2E,F). There are two main pathways to repair damaged DSBs: HR and NHEJ. Thus, the efficiencies of NHEJ and HR were quantitatively monitored in vivo via a CRISPR/Cas9-induced oligodeoxynucleotide (ODN) detection system as described in a previous report [22]. BPQDs treatment markedly decreased NHEJ activity in 786-O cells (Figure 2G) but did not affect HR repair (data not shown). These results suggest that BPQDs impair IR-induced DNA DSBs repair.

### 3.4. BPQDs Suppress DNA-PKcs Activity and Limit the Dynamics of DNA-PKcs at Damage Sites

**Figure 3:**
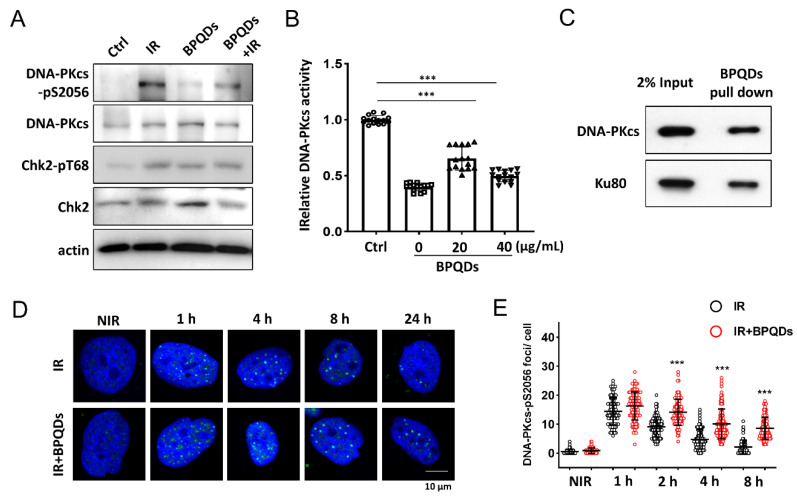
BPQDs inhibit DNA-PKcs activity. (**A**) Immunoblot showing DNA-PKcs pS2056 levels in BPQDs-treated or control 786-O cells at 30 min post 10 Gy IR. (**B**) The in vitro kinase activity of the purified DNA-PK complex was determined after incubation with the indicated dosage of BPQDs. (**C**) BPQDs were incubated with purified DNA-PK complex for 1 h. The BPQDs–protein complexes were centrifuged, washed, and analyzed by immunoblotting. (**D**) Representative IF images showing the foci of autophosphorylation of DNA-PKcs at Ser2056 in 786-O cells treated with 20 μg/mL BPQDs or PBS 2 h before 2 Gy IR (scale bar = 10 μm). (**E**) Number of DNA-PKcs-pSer2056 foci per cell at the indicated time points post-IR. *** *p* < 0.001.

DNA-PKcs is the key regulator of the NHEJ repair pathway [23]. We investigated whether BPQDs affect the activity of DNA-PKcs in response to IR. IR-induced autophosphorylation of DNA-PKcs on its Ser2056 site was significantly reduced in BPQDs-pretreated 786-O cells compared with the IR-only group (Figure 3A). We further investigated whether BPQDs affect DNA-PKcs activity in an in vitro system. BPQDs (20 and 40 μg/mL) were mixed with purified DNA-PK complexes, and DNA-PK activity was determined using p53 peptide as a substrate. The BPQDs significantly inhibited DNA-PKcs kinase activity in a dose-dependent manner (Figure 3B). The system was verified using treatment with the DNA-PKcs inhibitor Nu7441 (0.5 μM). The direct interaction between BP NPs and biological systems has attracted more attention. Here, we examined the possible association of BPQDs with the DNA-PK complex. As shown, purified DNA-PKcs and Ku80 could be well pulled down by BPQDs (Figure 3C). When we examined the foci of phosphorylated DNA-PKcs at Ser2056, an unexpected result showed that the frequency of pDNA-PKcs-Ser2056 foci after BPQDs treatment was significantly enhanced (Figure 3D,E). Autophosphorylation of DNA-PKcs at Ser2056 is essential for DNA-PKcs dissociation and the accessibility of its downstream factors at damage sites [8]. Therefore, BPQDs impair DNA-PKcs activity and trap it at the damage sites.

### 3.5. BPQDs Increase IR-Induced Mitotic Errors and Subsequent Micronuclei Formation

**Figure 4:**
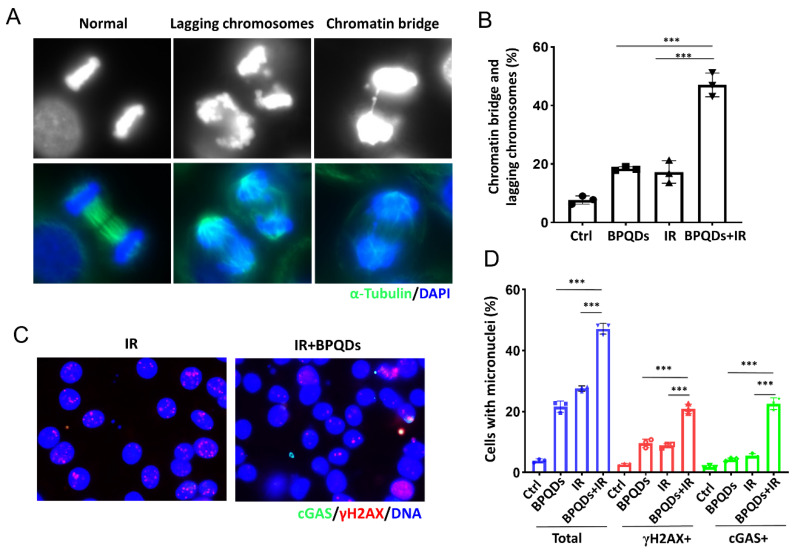
BPQDs increase IR-induced mitotic aberrations. (**A**) The 786-O cells were treated with BPQDs (20 μg/mL) and/or 4 Gy IR for 48 h. Cells were fixed and stained with α-tubulin (greed) and DAPI (blue). Representative immunofluorescence images are shown. The scale bar represents 10 µm. (**B**) Percentages of anaphase cells with chromatin bridges and lagging chromosomes (Data were generated from three independent experiments. *** *p* < 0.001). (**C**) The 786-O cells were treated with BPQDs (20 μg/mL) and/or 10 Gy IR for 48 h. Cells were fixed and stained with cGAS (greed), γH2AX (red), and DAPI (blue). Representative immunofluorescence images are shown. The scale bar represents 10 µm. (**D**) Percentages of anaphase cells with chromatin bridges and lagging chromosomes. *** *p* < 0.001.

Massive unrepaired DNA entering mitosis will lead to mitotic error and form lagging chromosomes and chromatin bridges. Emerging evidence shows that such errors in chromosome segregation trigger the generation of micronuclei (MNs), and the recognition of MNs by innate immune sensors, such as cGAS, leads to autoinflammation or antitumor immunity [24,25]. Here, we found that BPQDs markedly increased IR-induced lagging chromosomes and chromatin bridges in 786-O cells (Figure 4A,B). We further assessed whether BPQDs would exacerbate the formation of cGAS-positive micronuclei. As shown, BPQDs-treated RCC cells showed significantly increased formation of micronuclei as well as cGAS- and γH2AX-positive micronuclei in response to 10 Gy X-ray irradiation (22.6% in combined-treated cells versus 5.5% in IR-only cells, and 4.3% in BPQDs-treated cells) (Figure 4C,D).

### 3.6. BPQDs Sensitize RCC Cells to IR In Vivo

**Figure 5:**
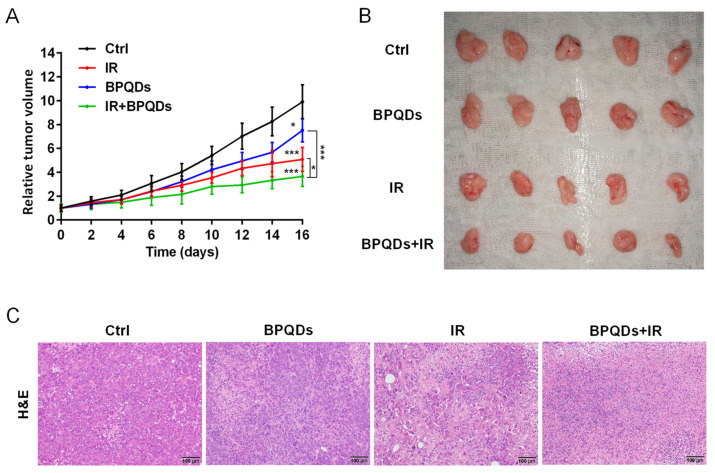
BPQDs and IR suppress tumor growth in a subcutaneous tumor model. (**A**) The 786-O tumor-bearing mice treated by PBS, IR, BPQDs, and IR + BPQDs. * *p* < 0.05, *** *p* < 0.001. (**B**) Tumor tissues from mice at the termination of the experiments. (**C**) Images of tumor tissue sections (H&E staining). Scale bar = 100 μm. *n* = 5 per treatment group.

To evaluate the potential radiosensitizing activity of BPQDs, we subcutaneously injected 786-O cells into athymic nude mice and recorded the volume of tumors. We found that the relative tumor volume was dramatically decreased when comparing mice with combined treatment (BPQDs + IR) versus either the BPQDs or IR-treated mice, suggesting the benefit of BPQDs on radiosensitization (Figure 5A,B). We also performed the histopathological examination of the dissected tumor tissues and found severe vacuolization and structural damage in the combination-treated versus the monotherapy and control groups (Figure 5C). These results suggested that BPQDs have the potential to radiosensitize the 786-O cells in in vivo.

## 4. Discussion

Our present study demonstrated that BPQDs can act as radiosensitizers in RCC because BPQDs-treated RCC cells exhibit sustained DNA damage signaling, which reflects defects in DNA DSB repair, particularly through NHEJ repair, and consequently enhance IR-induced apoptotic cell death. Furthermore, the results from the in vitro system showed that BPQDs can pull down the DNA-PK complex and inhibit DNA-PK kinase activity. Although our study showed that BPQDs impair IR-induced autophosphorylation of DNA-PKcs in RCC cells, the direct interaction between BPQDs and the DNA-PK complex in vivo and the translocation of BPQDs to the cell nucleus, especially on the damaged DNA ends, are still unanswered questions. Several studies have suggested that DNA-PK can be regulated by various cytoplasmic signaling pathways, including EGFR-Akt signaling [26,27], NF-κB signaling [28], and cytoskeleton-related signaling [29]. Our previous reports showed that BPQDs treatment leads to the stress fiber of microtubule [20], suggesting that BPQDs might regulate DNA-PKcs function through the cytoplasmic signaling pathway. BPQDs also suppress the deacetylase activity of HDAC1 in RCC cells. Histone deacetylases play multiple roles in regulating the DNA damage response, including NHEJ repair. HDAC1 and HDAC2 have been identified as upstream participants of NHEJ, at least in part by regulating the proper dynamics of NHEJ factors from damaged sites [30]. Here, we found that BPQDs trap phosphorylated DNA-PKcs at damaged sites. Whether BPQDs induce inappropriate disassembly of DNA-PKcs from DSBs sites by eliminating HDAC1 activity remains unclear and needs further investigation.

DNA-PKcs also functions as a key mitotic signaling kinase other than the DNA damage response [31,32]. Mitotic activation of DNA-PKcs is required for phosphorylation of downstream target factors, including Chk2 [33] and PLK1 [34,35]. DNA-PKcs-dependent Chk2-phosphorylation on its Thr68 site facilitates activation of the Chk2-BRCA1 pathway [33]. DNA-PKcs also colocalizes with PLK1, which is an essential kinase during mitosis progression [36], at the centrosome, and DNA-PKcs promotes the PLK1 activity-mediated G_2_/M transition [34]. Shao and colleagues showed that BP nanomaterials can exist on the centrosome to compromise centrosome integrity by deactivating the PLK1 activity [19]. The BP nanomaterials interact with PLK1, leading to its aggregation and restricting the recruitment of PLK1 to centrosomes. The BPQDs may block the interaction between DNA-PKcs and PLK1, and disrupt DNA-PKcs-mediated activation of PLK1. Our recent study revealed that DNA-PKcs associates with HDAC6 and modulates HDAC6-mediated deacetylation of HSP90, which is important to maintain the protein stability of the mitotic kinase Aurora A [37]. Aurora A plays essential roles in regulating mitotic spindle formation, and inhibition of Aurora A leads to failure of chromosome congression at metaphase and lagging chromosomes and chromatin bridges in anaphase [38]. Consistent with this, our present data showed that BPQDs treatment enhanced IR-induced lagging chromosomes and chromatin bridges in RCC cells, indicating that BPQDs may also influence HDAC6-HSP90 signaling via suppression of DNA-PKcs.

Cytosolic self-DNA, such as micronuclei, can be generated by mitosis error following DNA damage in mammalian cells, triggering cGAS-STING-dependent inflammatory signaling [24,25]. Most recent work identified that DNA-PKcs phosphorylates cGAS and inhibits its enzymatic activity. DNA-PKcs deficiency enhances the cGAS-mediated innate immune response [38]. The BPQDs suppress the kinase activity of DNA-PKcs, impair DNA DSBs repair efficiency, and disrupt the mitotic spindle structure. In line with this notion, we observed that BPQDs-pretreated RCC cells exhibit an increased number of IR-induced micronuclei and an elevated amount of cGAS localization to micronuclei, suggesting that BPQDs may have the potential to enhance IR-induced innate immunity in RCC cells. RCC is traditionally considered to be resistant to conventionally fractionated radiotherapy with the dose 1.8–2.1 Gy per fraction [39]. Modern technological advances in radiation oncology have increased the efficacy of radiotherapy, allowing higher dosage delivery to tumor, and leading to effective management of cancer patients [40]. Recent studies showed that the application of stereotactic body radiotherapy (SBRT) was associated with better local control of metastatic RCC [41,42,43]. These studies show that RCC can no longer simply be recognized as radioresistant, and more studies are necessary for exploring the combination of SBRT with other therapy strategies [44]. Therefore, development of target-based radiosensitization strategies to sensitize cancer cells to RT become attractive therapeutic strategy for the clinical benefit of RCC patients. Significant evidence has revealed the potential of DNA-PKcs in cancer development; thus, various anti-DNA-PKcs strategies have been proposed as either monotherapy or in combination with chemo- and radiotherapy [45]. Here, we found that BPQDs can inhibit the kinase activity of DNA-PKcs and radiosensitize RCC cells in vivo and in vitro.

## 5. Conclusions

In summary, we found that BPQDs inhibit DNA-PKcs activity and impair DNA-PKcs-mediated NHEJ DNA DSBs repair, resulting in sustained DNA damage in response to IR. BPQDs enhances IR-induced suppression of RCC xenografts growth in vivo, pointing toward a promising BPQDs-based targeted cancer therapy.

## Data Availability

Not applicable.

## Supplementary Materials

The following supporting information can be downloaded at: https://www.mdpi.com/article/10.3390/cells11101651/s1, Figure S1: TEM and AFM images of BPQDs; Figure S2. BPQDs treatment sensitizes RCC cells to irradiation; Figure S3. BPQDs decrease the capacity of DNA DSBs and enhance IR-induced apoptosis in A498 cells; Table S1: Characterization of BPQDs.
