# Supplementary material for: Black Phosphorus Quantum Dots Enhance the Radiosensitivity of Human Renal Cell Carcinoma Cells through Inhibition of DNA-PKcs Kinase
Source: Cells. 2022 May 16;11(10):1651. doi: 10.3390/cells11101651 (PMC9139844; doi:10.3390/cells11101651)
Supplement: Supplementary file 1 [file cells-11-01651-s001.zip › cells-1668767-supplementary.pdf]

Supporting information

Black Phosphorus Quantum Dots Enhance the Radiosensitivity of Human Renal Cell Carcinoma Cells through Inhibition of DNA-PKcs Kinase

Yue Lang[1,2], Xin Tian[1], Hai-Yue Dong[1], Xiang-Xiang Zhang[1], Lan Yu[3], Ming Li[1], Meng-Meng Gu[4], Dexuan Gao[5], Zeng-Fu Shang[1]

[1] State Key Laboratory of Radiation Medicine and Protection, School of Radiation Medicine and Protection, Medical College of Soochow University, Collaborative Innovation Center of Radiation Medicine of Jiangsu Higher Education Institutions, Soochow University, Suzhou 215123, China.

[2] The Second Affiliated Hospital of Xuzhou Medical University, Xuzhou 221000, Jiangsu, China.

[3] Suzhou Digestive Diseases and Nutrition Research Center, The Affiliated Suzhou Hospital of Nanjing Medical University, Suzhou 215008, China.

[4] Department of Nuclear Medicine, The Affiliated Suzhou Hospital of Nanjing Medical University, Suzhou 215002, China.

[5] Department of Urology, Shandong Provincial Hospital Affiliated to Shandong First Medical University, Jinan 250021, China.

Other methods

Synthesis and Characterization of BPQDs

Bulk BP crystals (99.998%) were mixed with NMP (99.5%, anhydrous) and maintained for 12 h at 140 °C under vigorous stirring and N_2 protection. Afterwards, this mixture was centrifuged at 12,000 rpm for 20 min, and the supernatant solution was collected for further characterization and application. The morphology and thickness of BPQDs were detected using transmission electron microscopy (TEM, Tecnai G-20, FEI) and atomic force microscopy (AFM, Dimension Icon, Bruker), respectively. The hydrodynamic size and zeta potential of BPQDs were measured using a particle analyzer (Zetasizer Nano-ZS, Malvern).

Table S1 Characterization of BPQDs.

Characterization	Condition	Results
Zeta potential	DI water	-24.3 ± 0.8 mV
Hydrodynamic size	DI water	11.7 ± 1.5 nm
Zeta potential	PBS	-16.9 ± 0.3 mV
Hydrodynamic size	PBS	26.5 ± 2.3 nm

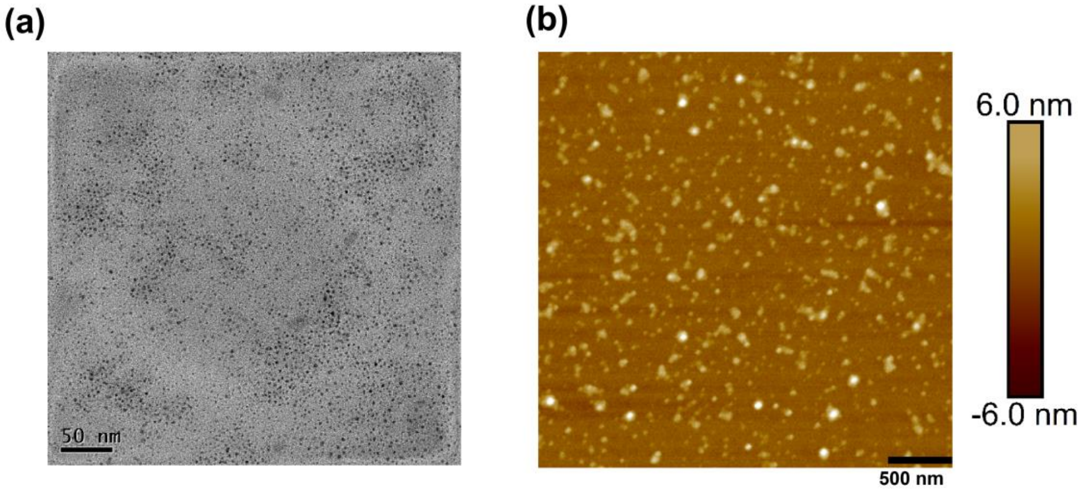

Figure S1. (a) TEM and (b) AFM images of BPQDs.

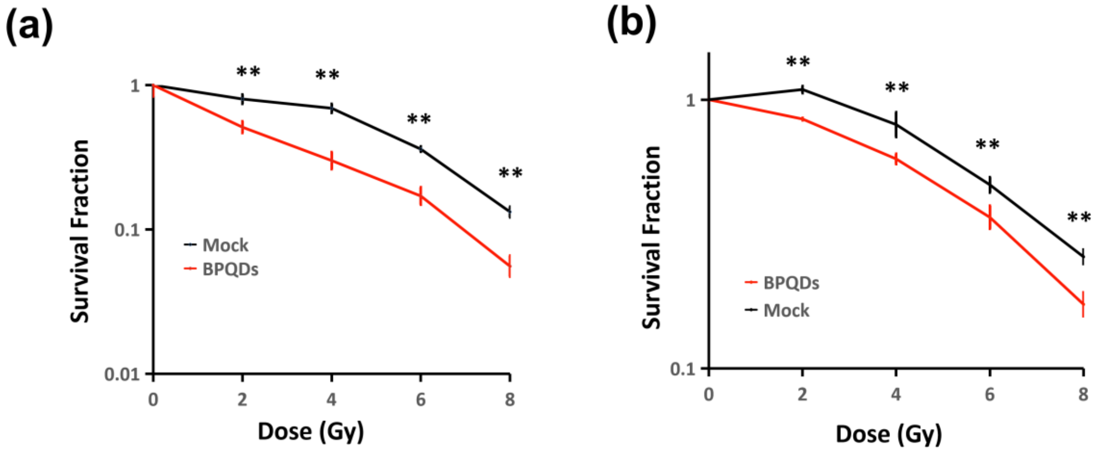

Figure S2. BPQDs treatment sensitizes RCC cells to irradiation. (a) 786O and (b) A498 cells were subjected to indicated dose of X-ray irradiation in combination with or without BPQDs treatment (20 μg/mL, 12 h before IR), and were analyzed for clonal survival ability, $^{**}P < 0.01$.

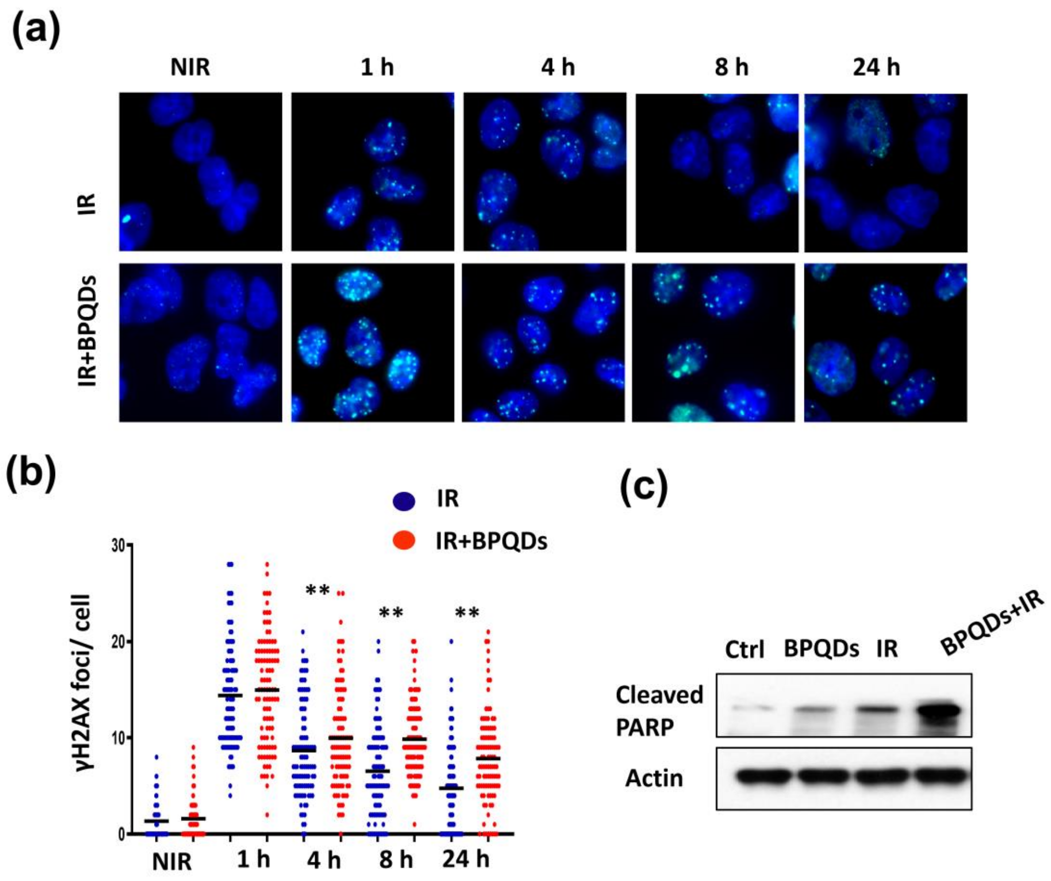

Figure S3. BPQDs decrease the capacity of DNA DSBs and enhance IR-induced apoptosis in A498 cells. (**a**) Representative IF images showing γH2AX foci in A498 cells treated with 20 μg/mL BPQDs or PBS 2 h before 2 Gy IR . (**b**) Number of γH2AX foci per cell at the indicated time points post-IR ($^{**}p < 0.01$). (**c**) A498 cells were exposed to 20 μg/mL BPQDs along or in combination with 5 Gy IR for 24 h and subjected to immunoblotting with anti-Cleaved PARP (Asp214), and anti-Actin antibodies.
